# Compromised Grid-Cell-like Representations in Old Age as a Key Mechanism to Explain Age-Related Navigational Deficits

**DOI:** 10.1016/j.cub.2018.02.038

**Published:** 2018-04-02

**Authors:** Matthias Stangl, Johannes Achtzehn, Karin Huber, Caroline Dietrich, Claus Tempelmann, Thomas Wolbers

**Affiliations:** 1 German Center for Neurodegenerative Diseases (DZNE), Aging & Cognition Research Group, Leipziger Str. 44, 39120 Magdeburg, Germany; 2 Otto-von-Guericke-University Magdeburg, Department of Neurology, Leipziger Str. 44, 39120 Magdeburg, Germany; 3 Center for Behavioral Brain Sciences, Universitätsplatz 2, 39106 Magdeburg, Germany

**Keywords:** aging, human, spatial navigation, grid cells, path integration, entorhinal cortex, fMRI

## Abstract

A progressive loss of navigational abilities in old age has been observed in numerous studies, but we have only limited understanding of the neural mechanisms underlying this decline [1]. A central component of the brain’s navigation circuit are grid cells in entorhinal cortex [2], largely thought to support intrinsic self-motion-related computations, such as path integration (i.e., keeping track of one’s position by integrating self-motion cues) [3, 4, 5, 6]. Given that entorhinal cortex is particularly vulnerable to neurodegenerative processes during aging and Alzheimer’s disease [7, 8, 9, 10, 11, 12, 13, 14], deficits in grid cell function could be a key mechanism to explain age-related navigational decline. To test this hypothesis, we conducted two experiments in healthy young and older adults. First, in an fMRI experiment, we found significantly reduced grid-cell-like representations in entorhinal cortex of older adults. Second, in a behavioral path integration experiment, older adults showed deficits in computations of self-position during path integration based on body-based or visual self-motion cues. Most strikingly, we found that these path integration deficits in older adults could be explained by their individual magnitudes of grid-cell-like representations, as reduced grid-cell-like representations were associated with larger path integration errors. Together, these results show that grid-cell-like representations in entorhinal cortex are compromised in healthy aging. Furthermore, the association between grid-cell-like representations and path integration performance in old age supports the notion that grid cells underlie path integration processes. We therefore conclude that impaired grid cell function may play a key role in age-related decline of specific higher-order cognitive functions, such as spatial navigation.

## Highlights

• Grid-cell-like representations in human entorhinal cortex are compromised in old age
• This effect is predominantly driven by a lack of representational stability over time
• Path integration ability in old age is associated with grid-cell-like representations

## Teaser

Stangl et al. found that human grid-cell-like representations are compromised in old age and that reduced magnitudes of grid-cell-like representations are predictive of age-related path integration deficits. Impaired grid cell function, therefore, may provide a mechanistic explanation for a decline of navigational functions in old age.

## Results and Discussion

### Reduced Grid-Cell-like Representations in Older Adults

**Figure 1:**
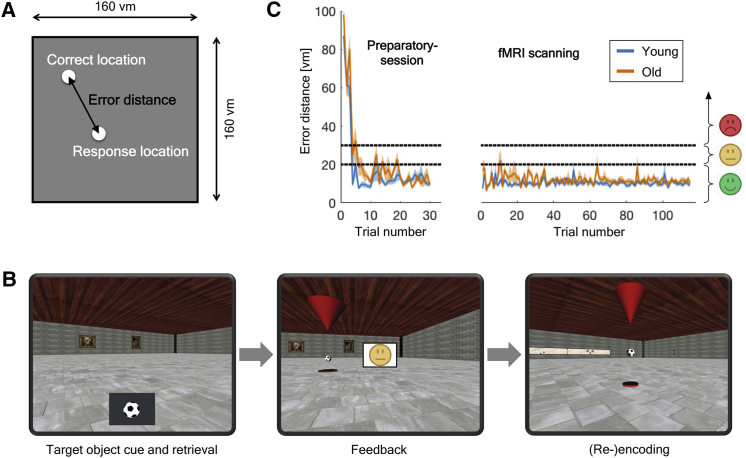
Object-Location Memory Task (A) During fMRI scanning, participants performed multiple trials of an object-location memory task in a virtual environment (160 × 160 virtual meters [vm]), which contained three target objects. In each trial, participants had to navigate to the location of one cued target object. Task accuracy was expressed for each trial by the “error distance,” which was calculated as the Euclidean distance between the target object’s correct location and the participant’s response. (B) Example trial of the object-location memory task: First, all target objects disappeared, and a cue image of one target object was shown (e.g., a soccer ball). Participants navigated to the position of the cued target object and confirmed their choice of location with a button press. A smiley face provided feedback as to the accuracy of the response. Participants were instructed to aim for green smiley face feedback (error distances <20 vm) and avoid yellow smiley faces (error distances 20–30 vm) or red smiley faces (error distances >30 vm). If the error distance was larger than 20 vm, the target object reappeared and had to be collected, allowing for (re-)encoding of its correct location. After each trial, the participant was automatically transported to a random position within the environment before the next trial started. (C) Task accuracy in the object-location memory task. After having learned the target object’s locations in the preparatory session (left panel), both young and older adults were able to perform the task with required accuracy during fMRI scanning (right panel). Blue and orange lines indicate group mean ± SEM; black dashed lines indicate error distance thresholds for different smiley face feedback. Plots show the first 30 trials of the preparatory session and 115 trials for fMRI scanning, which are the minimum numbers of trials that all participants completed.

Grid-cell-like representations in humans, reflecting the 6-fold symmetric grid cell firing pattern routinely seen in electrophysiology studies, can be measured by fMRI during navigation in virtual environments [15, 16, 17]. Here, healthy young and older adults performed an object-location memory task in a virtual environment, which contained three target objects (Figures 1A and 1B). All participants received extensive pre-training in a preparatory session before scanning (see STAR Methods) to ensure that they were able to continuously retrieve all target object locations with an error distance below 20 virtual meters (vm). This procedure proved successful, because the average error distance across all trials during fMRI scanning was below 20 vm for all participants, showing that they were able to perform the task with the required accuracy (Figure 1C).

For the analysis of grid-cell-like representations, fMRI data were split in two halves, using the first half to estimate voxel-wise grid orientations in bilateral entorhinal cortex and then testing these orientations on the other half of the data in order to quantify the magnitude of each individual participant’s grid-cell-like representations (see STAR Methods).

**Figure 2:**
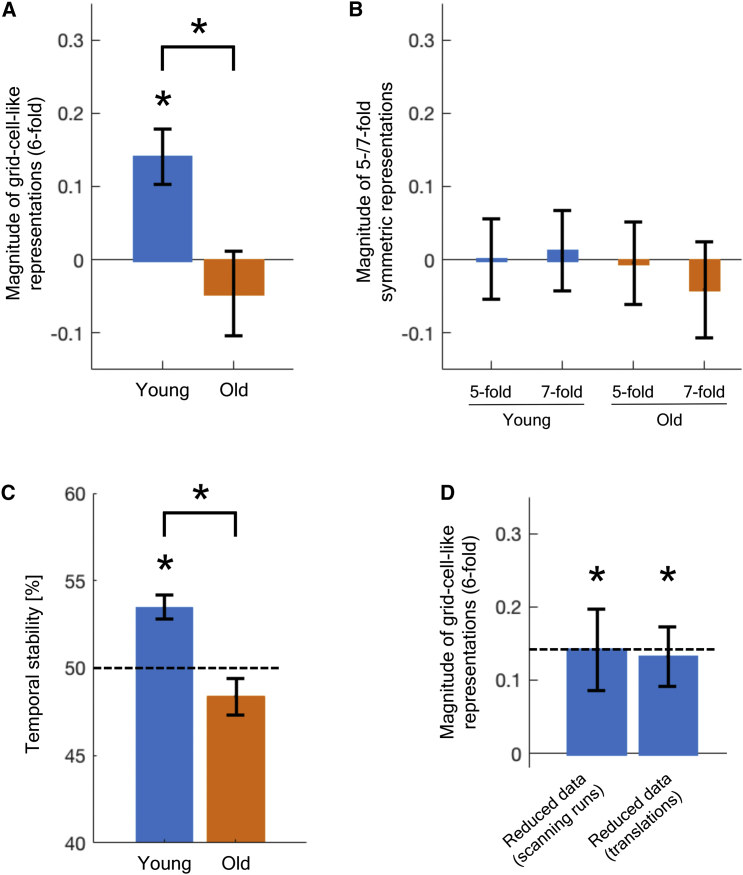
Grid-Cell-like Representations in Entorhinal Cortex of Young and Older Adults (A) Higher magnitude of grid-cell-like representations in young relative to older adults. The effect of grid-cell-like representations in older adults was not significantly different from zero. (B) In control analyses for different symmetrical models (5-fold/7-fold), representational magnitudes were not significantly different from zero, either for young or for older adults. (C) Lower temporal stability of grid-cell-like representations in older as compared to young adults. Dashed line indicates 50% chance level. Temporal stability scores were significantly different from chance level in young, but not in older, adults (young: t_19_ = 4.93, p < 0.001; old: t_20_ = −1.55, p = 0.136). (D) Significant grid-cell-like representations in young adults for models with reduced data, both when the duration of scanning runs (left bar) or the duration of individual translation events (right bar) was reduced. Dashed line indicates mean grid-cell-like representation magnitude of young adults for the full dataset. Error bars indicate SEM; ^∗^p < 0.05; units of representational magnitudes are parameter estimates. See also Figures S1 and S2 and STAR Methods.

We found significant grid-cell-like representations in entorhinal cortex of young adults (Figure 2A). Specifically, this effect was only significant for the 6-fold symmetrical model (t_19_ = 3.71; p = 0.002), whereas effects in control analyses applying symmetrical models with different periodicities (Figure 2B) were not significantly different from zero (5-fold: t_19_ = 0.02, p = 0.983; 7-fold: t_19_ = 0.23, p = 0.822). Furthermore, we found that the magnitude of grid-cell-like representations in older adults was significantly reduced compared to young adults (t_39_ = −2.66; p = 0.011). In fact, we did not find any significant effect of grid-cell-like representations in older adults, either for the 6-fold symmetrical model (t_20_ = −0.79; p = 0.438) or for the control models testing for other periodicities (5-fold: t_20_ = −0.08, p = 0.940; 7-fold: t_20_ = −0.62, p = 0.544).

Reduced grid-cell-like representations in older adults could result from a lack of temporal stability (i.e., stability of voxel-wise grid orientations over time) or from insufficient spatial stability (i.e., homogeneity of voxel-wise grid orientations across all entorhinal cortex voxels) [16, 18]. To further specify which of these two factors was driving the result of reduced grid-cell-like representations, we calculated indicators of temporal and spatial representational stability for each participant (see STAR Methods) and compared these between age groups. We found significantly reduced temporal stability of grid orientations in older adults (t_39_ = 4.01; p < 0.001; Figure 2C). This result was also confirmed by a separate analysis showing that changes in estimated voxel-wise grid orientations over time were significantly larger in older than in young adults (t_39_ = −4.29; p < 0.001). On the contrary, spatial stability scores did not differ between age groups (t_39_ = 0.91; p = 0.370; Figure S2A). Given that the lack of a difference in spatial stability could result from spatial smoothing of the fMRI time series, we also calculated spatial stability scores for unsmoothed data, but again, we did not find a significant difference between age groups (t_39_ = 0.53; p = 0.597).

These results indicate that grid-cell-like representations in entorhinal cortex are compromised in old age and that this effect is predominantly driven by a lack of temporal representational stability. Recently, grid-cell-like representations have been investigated also in young adults at increased genetic risk for Alzheimer’s disease [16]. We note that this Alzheimer’s risk group showed a remarkably similar pattern of data (i.e., both reduced magnitudes of grid-cell-like representations and reduced temporal stability) as compared to the older adults in the present study. Consequently, the present data suggest that impaired grid cell function is not only related to pathological neurodegenerative processes but also occurs during normal healthy aging.

Whereas no previously reported study has investigated changes of the grid cell system in old age, there is evidence that hippocampal place cells also show temporal instability of spatial representations in old rats [19, 20]. Given the strong interconnections between entorhinal cortex and hippocampus [21] and previous findings showing coordinated temporal dynamics of entorhinal grid cells and hippocampal place cells [22], reduced temporal stability in the firing of spatially tuned neurons may be a common neuronal mechanism underpinning age-related deficits in computations of self-position.

There is evidence that entorhinal cortex receives input from the head direction system [23, 24, 25], and it has been reported that the firing of grid cells is modulated by head direction [26]. It is therefore possible that compromised grid-cell-like representations might be driven by impairments in head direction signals. To date, however, no single human or animal study has investigated age-related changes in the head direction system; therefore, this remains an important goal for future studies.

### Control Analyses

Cognitive aging is accompanied by many behavioral and neurophysiological changes that could serve as alternative explanations for our finding of reduced grid-cell-like representations in older adults. For example, we found that, relative to young adults, older adults spent more time per trial standing still (t_39_ = −7.55; p < 0.001) and rotating (t_39_ = −4.93; p < 0.001) in the virtual environment during the object-location memory task. This in turn led to a lower total duration of translation phases in older adults (young: 1,810 ± 123 s; old: 1,451 ± 200 s; t_39_ = 6.88; p < 0.001). In order to check whether the age difference in grid-cell-like representations might be driven by the different amounts of data between age groups, we re-ran the analysis of grid-cell-like representations for young adults, using only a proportion of their translation data to match the data of older adults. Data reduction in young adults was implemented in two different ways, by shortening scanning runs and shortening translation durations, respectively (see STAR Methods). The results of these control analyses mirrored the original results using the full dataset (Figure 2D): in both reduced datasets of young adults, we found a significant effect of grid-cell-like representations (shortened scanning runs: t_19_ = 2.53, p = 0.021; shortened translations: t_19_ = 3.25, p = 0.004), and the magnitude of grid-cell-like representations was not significantly different from the full dataset (shortened scanning runs: t_19_ = −0.01, p = 0.992; shortened translations: t_19_ = 0.46, p = 0.653) but remained significantly higher relative to older adults (shortened scanning runs: t_39_ = −2.32, p = 0.026; shortened translations: t_39_ = −2.49, p = 0.017). We therefore conclude that the difference in the amount of data did not drive the detected age difference in grid-cell-like representations, because this effect still persisted when modeling an identical amount of translation data for both age groups.

Second, the average error distance in the object-location memory task was slightly lower for young than for older adults (young: 10.3 ± 1.8 vm; old: 12.0 ± 2.6 vm; t_39_ = −2.41; p = 0.021). However, error distances and magnitudes of grid-cell-like representations were not correlated within either of the two groups (young: r = −0.19, p = 0.417; old: r = −0.02, p = 0.934), and an analysis of covariance confirmed that magnitudes of grid-cell-like representations were still significantly different between age groups when controlled for individual error distances (F_1,38_ = 5.28; p = 0.027). Therefore, it is unlikely that differences in task accuracy were driving the age difference in grid-cell-like representations.

Third, we did not find a difference between age groups in entorhinal cortex volume (see STAR Methods) that could potentially account for the observed difference in grid-cell-like representations (t_39_ = 1.38; p = 0.177; Figure S2B). Also, there was no correlation between individual entorhinal cortex volume and grid-cell-like representation magnitude, either within young or older adults (young: r = −0.14, p = 0.554; old: r = 0.30, p = 0.186), and grid-cell-like representation magnitudes were significantly different between age groups in an analysis of covariance controlling for entorhinal cortex volume (F_1,38_ = 6.01; p = 0.019).

Fourth, signal quality in entorhinal cortex could not account for differences in grid-cell-like representations between young and older adults, as the temporal signal-to-noise ratio (tSNR) did not significantly differ between age groups (t_39_ = 1.63; p = 0.112; Figure S2C; see STAR Methods), and magnitudes of grid-cell-like representations were not significantly correlated with tSNR within each group (young: r = 0.17, p = 0.484; old: r = 0.07, p = 0.776). Moreover, magnitudes of grid-cell-like representations remained significantly different between age groups in an analysis of covariance controlling for tSNR (F_1,38_ = 5.71; p = 0.022).

Finally, we found that older adults made more linear and angular head movements during scanning than young adults (linear: t_39_ = −3.97, p < 0.001; angular: t_39_ = −3.26, p = 0.002; Figure S2D; see STAR Methods). However, head movement is unlikely to account for group differences in grid-cell-like representations, as it did not correlate with grid-cell-like representation magnitudes within either of the two groups (young/linear: r = −0.16, p = 0.496; young/angular: r = −0.05, p = 0.842; old/linear: r = −0.05, p = 0.829; old/angular: r = −0.01, p = 0.976), and an analysis of covariance confirmed that magnitudes of grid-cell-like representations were still significantly different between age groups when controlled for both linear and angular head movement (F_1,38_ = 6.91; p = 0.012).

### Path Integration Deficits in Older Adults

**Figure 3:**
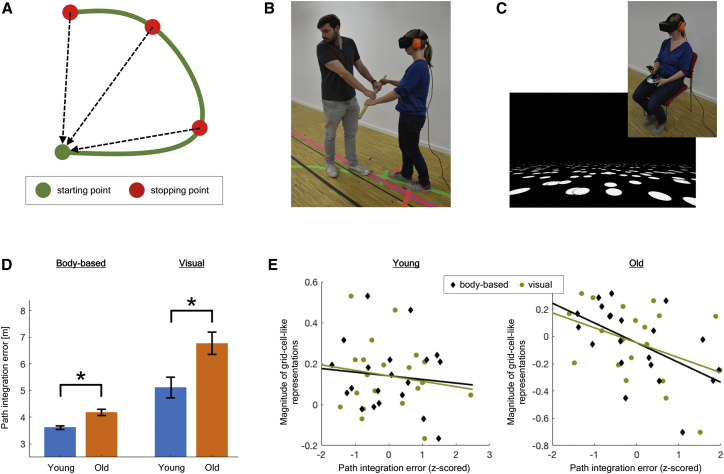
Path Integration Performance and Association with Entorhinal Grid-Cell-like Representations (A) Example path from top-down perspective. There were three stopping points along each path. While walking along a path, participants had to stop at each stopping point and estimate the direct distance and orientation to the path’s starting point (black dashed arrows). (B) In the “body-based” modality, the head-mounted display showed no visual input, so that participants experienced only body-based cues during movement along the path. Participants held a wooden stick and were guided by the experimenter along the path. At each stopping point, the distance to the starting point had to be estimated verbally in meters and centimeters and participants turned their body on the spot to indicate the orientation to the starting point. (C) In the “visual” modality, participants sat stationary on a chair. The head-mounted display showed a floor texture consisting of white “limited lifetime dots” on a black ground plane, which provided optic flow information while preventing use of fixed reference points (see STAR Methods). Automated movement along each path was shown from first-person perspective, and on each stopping point, participants estimated the distance to the path’s starting point verbally in meters and centimeters and indicated the presumed orientation by turning their view in the virtual environment to the left or right using a joystick. (D) Higher path integration errors of older adults, both in the body-based and the visual modality. Error bars indicate SEM; ^∗^p < 0.05. (E) In young adults (left), the magnitude of grid-cell-like representations was not associated with path integration errors in either the body-based or the visual modality. In older adults (right), higher magnitudes of grid-cell-like representations were associated with lower path integration errors. Path integration errors were z-scored for display purposes. See also Figure S3 and STAR Methods.

To investigate potential links between grid-cell-like representations and path integration abilities, we measured each participant’s individual performance in an independent behavioral path integration experiment. Participants had to keep track of their own position during movement along pre-defined curved paths (Figure 3A). This path integration task was implemented in two different modalities: the “body-based” modality (Figure 3B) provided only body-based self-motion cues during movement, whereas in the “visual” modality (Figure 3C), only visual cues could be used. There were eight different pre-defined paths (Figure S3C), and each path was performed twice per modality. Path integration error was first calculated for each stopping point of a path separately (see STAR Methods) and then accumulated across all paths of the same modality in order to calculate an individual modality-specific measure of path integration performance per participant.

Relative to young adults, older adults showed a reduced performance in the path integration task (Figure 3D). In both the body-based and the visual modality, path integration errors of older adults were significantly higher than those of young adults (body based: t_39_ = −4.14, p < 0.001; visual: t_39_ = −2.89, p = 0.006). These results demonstrate a modality-independent path integration deficit in old age. More specifically, older adults showed a deficit in computations of self-position, independent of whether path integration was based on body-based or visual self-motion cues. This is widely in line with results from previous studies that reported age-related path integration impairments for different sensory modalities [27, 28, 29, 30].

### Association between Grid-Cell-like Representations and Path Integration Performance in Older Adults

When combining data from both the fMRI and the path integration experiment, we found that, within the group of older adults, those participants with a higher magnitude of grid-cell-like representations showed lower path integration errors (Figure 3E). This was indicated by a significant correlation between grid-cell-like representation magnitudes of older adults and their path integration errors in the body-based modality (r = −0.54; p = 0.011). A similar pattern was found in older adults also for the visual modality, although this correlation did not reach statistical significance level (r = −0.41; p = 0.065). In young adults, there was no correlation between grid-cell-like representation magnitudes and path integration errors, either in the body-based (r = −0.10; p = 0.660) or the visual modality (r = −0.16; p = 0.511).

Given the significant association between magnitudes of grid-cell-like representations and path integration performance in the body-based modality, we split up the group of older adults based on their median body-based path integration error and further analyzed grid-cell-like representations in those older adults who showed low path integration errors (n = 10). In this subgroup, we found a significant magnitude of grid-cell-like representations (t_9_ = 3.55; p = 0.006), whereas control models testing for other periodicities were not significantly different from zero (5-fold: t_9_ = −0.78, p = 0.454; 7-fold: t_9_ = −0.48, p = 0.640). Moreover, their magnitude of grid-cell-like representations was not significantly different compared to young adults (t_28_ = 0.06; p = 0.954). We therefore conclude that grid-cell-like representation magnitudes in older adults who showed high path integration performance appear to be similar to those of the young adult group (Figure S2E).

Next, we applied multiple linear regression on the data of older adults in order to compare the predictive value of grid-cell-like representations to a range of other factors that might potentially explain variability in path integration performance. In addition to grid-cell-like representations and individual demographic variables, this included a range of test scores from a neuropsychological test battery (see STAR Methods). Results of this regression analysis confirmed a significant link between grid-cell-like representations and both body-based as well as visual path integration performance, whereas no other demographic or neuropsychological factor could significantly predict path integration performance (Table 1).

**Table 1:** Multiple Linear Regression to Predict Path Integration Performance of Older Adults in Body-Based and Visual Modality

Body-Based Modality				
Predictor	β	t	p	Sign.
Grid-cell-like representations	0.069	2.717	0.022	p < 0.05
Age	−0.002	−1.137	0.282	ns
Sex: male	0.023	1.784	0.105	ns
Self-reported spatial abilities (SBSOD^a^)	0.003	0.472	0.647	ns
Spatial working memory (CORSI^b^)	0.005	1.724	0.115	ns
Spatial attention (TAP^c^)	0.000	0.832	0.425	ns
Working memory (TAP^d^)	0.000	−0.550	0.595	ns
Processing speed (DSST^e^)	0.001	1.146	0.279	ns
Cognitive status (MoCA^f^)	0.004	1.431	0.183	ns

**Visual Modality**				

Predictor	β	t	p	Sign.

Grid-cell-like representations	0.155	2.517	0.031	p < 0.05
Age	0.007	1.555	0.151	ns
Sex: male	−0.023	−0.729	0.483	ns
Self-reported spatial abilities (SBSOD^a^)	0.006	0.425	0.680	ns
Spatial working memory (CORSI^b^)	0.012	1.559	0.150	ns
Spatial attention (TAP^c^)	0.000	0.521	0.613	ns
Working memory (TAP^d^)	0.000	−0.761	0.464	ns
Processing speed (DSST^e^)	0.000	0.116	0.910	ns
Cognitive status (MoCA^f^)	0.009	1.280	0.229	ns

ns, not significant.

a Santa Barbara Sense of Direction Scale

b Corsi block-tapping test

c Testbattery for Attentional Performance (TAP): subtest “visual scanning”

d Testbattery for Attentional Performance (TAP): subtest “working memory”

e Digit symbol substitution test (WAIS-IV)

f Montreal Cognitive Assessment

Whereas animal studies and theoretical models have long suggested that path integration is a key function of the grid cell system, direct empirical evidence for this claim is scarce [4, 5, 6, 25, 31, 32, 33, 34, 35, 36]. Our data show that the magnitude of grid-cell-like representations in the human entorhinal cortex is linked to path integration performance in old age and therefore further strengthens the hypothesis that grid cell function underlies path integration processes.

It is unclear, however, why we did not find an association between grid-cell-like representations and path integration performance in young adults. Potentially, this can be explained by additional processes, besides grid cell function, that may also contribute to path integration. For example, previous neuroimaging studies have demonstrated contributions of hippocampal and prefrontal computations [37, 38, 39]. Variability in these processes could explain variations in path integration performance when the grid cell system is intact and able to provide appropriate positional computations (as assumed to be seen in young adults). In compromised grid cell systems (as more likely to be present in older adults), however, fundamental computations of positional information might be impaired, resulting in a direct relationship between the degree of impairment and path integration performance. To further test this assumption, we split up the group of young adults using cluster analysis to identify three subgroups of participants with low, middle, and high magnitudes of grid-cell-like representations (see STAR Methods). Within the group of young adults with low grid-cell-like representation magnitudes (n = 5), we found that their magnitudes of grid-cell-like representations were significantly correlated with path integration errors in the body-based modality (r = −0.89; p = 0.045), whereas no correlation could be found for the group of young adults who had middle (n = 12; r = 0.43; p = 0.159) or high (n = 3; r = 0.54; p = 0.634) magnitudes of grid-cell-like representations. In the visual modality, the correlation values for the low group were also numerically stronger but failed to reach statistical significance (low: r = −0.59, p = 0.298; middle: r = −0.22, p = 0.490; high: r = −0.28, p = 0.820). Whereas these analyses need to be treated with caution due to the small group sizes, they support the theoretical assumption that an association between grid-cell-like representations and path integration performance is only seen when grid-cell-like representations are compromised.

### Conclusions

We have demonstrated here that grid-cell-like representations in the human entorhinal cortex are compromised in old age. Specifically, grid-cell-like representations were less stable over time in older as compared to young adults. Given that grid cells play a central role in higher order cognitive functions, these findings imply that deficient grid cell firing might be a key mechanism that could mediate cognitive deficits in old age. Not only does this provide important insights into age-related neurophysiological changes, but it is also a necessary precondition for designing efficient interventions and therapeutic approaches to counteract age-related cognitive decline. Moreover, we found that individual magnitudes of grid-cell-like representations in older adults could explain individual performance differences in path integration. This finding further strengthens the hypothesis that computations of self-position during movement rely on entorhinal grid cell function, as has been suggested previously [3, 4, 5]. In addition, we have shown that the prognostic value of grid-cell-like representations for predicting path integration performance in older adults clearly exceeds the prognostic value of a variety of standard neuropsychological tests and demographic variables. Together, these findings lay the foundation for future studies aiming to further explore whether human grid-cell-like representations in entorhinal cortex could potentially serve as a biomarker for integrity of the grid cell system and entorhinal cortex function. Because both neurophysiological changes in entorhinal cortex [7, 8, 9, 10, 11, 12, 14] and behavioral changes in navigational functions [40, 41, 42] are among the earliest symptoms of neurodegenerative processes like Alzheimer’s disease, such a prognostic measure could also facilitate early detection of dementia and other neurodegenerative disorders. Furthermore, as grid cells have been discussed to not only provide the neuronal basis for navigational functions but also dimensional coding in non-spatial domains [43, 44], future investigations will show whether changes in the grid cell system might explain not only spatial navigation deficits but also age-related decline in other cognitive domains.

## STAR★Methods

### Key Resources Table

**Table:** 

REAGENT or RESOURCE	SOURCE	IDENTIFIER
**Software and Algorithms**		

MATLAB 2016b	The MathWorks, Natick, MA, USA	https://www.mathworks.com/products/new_products/release2016b.html
Curve Fitting Toolbox for MATLAB 2016b	The MathWorks, Natick, MA, USA	https://www.mathworks.com/products/curvefitting.html
Statistics and Machine Learning Toolbox for MATLAB 2016b	The MathWorks, Natick, MA, USA	https://www.mathworks.com/products/statistics.html
Statistical Parametrical Mapping Toolbox (SPM12)	Functional Imaging Laboratory, University College London, UK	http://www.fil.ion.ucl.ac.uk/spm/software/spm12/
The Grid Code Analysis Toolbox (GridCAT) v1.04	[18]	https://www.nitrc.org/projects/gridcat

### Contact for Reagent and Resource Sharing

Further information and requests for resources should be directed to and will be fulfilled by the Lead Contact, Matthias Stangl (matthias.stangl@dzne.de).

### Experimental Model and Subject Details

41 healthy humans took part in this study. The group of young adults consisted of 20 participants (10 woman, 10 men) aged between 19 and 30 years (M = 24.5, SD = 3.3 years), whereas the group of older adults consisted of 21 participants (11 woman, 10 men) aged between 63 and 81 years (M = 69.3, SD = 4.8 years). Only participants with no reported history of neurological or psychiatric disease and no reported motor deficits during normal walking or standing took part in this study. All participants reported right-handedness and had normal or corrected-to-normal eyesight.

Informed consent was obtained from all participants in writing before the measurements, and the experiment received approval from the Ethics Committee of the University of Magdeburg.

Prior to the study, all participants underwent the Montreal Cognitive Assessment (MoCA) screening tool for mild cognitive impairment [45]. Participants who did not exceed a MoCA cut-off score of 23 (following Luis et al. [46]) were excluded from the study and did not participate in any further measurements. Participants also completed an image-recognition task, which is not discussed further in this paper.

### Method Details

#### Object-location memory task

Participants performed an object-location memory task in a virtual environment (Figure 1), using an MR-compatible joystick (Tethyx, Current Designs, http://www.curdes.com). Given that older adults often have less experience in using computers and gaming [47], the task was designed to be relatively simple in order to allow for similar task performance between young and older adults. In this task, participants were asked to complete multiple trials in a square virtual room with the dimensions 160 × 160 virtual meters (vm), in which three target-objects (ball, plant, trash bin) were placed in random locations. Each trial had the following structure: At the start, all target-objects disappeared, and a cue image of one target-object was shown at the bottom of the screen. Participants were asked to navigate to the position of the cued target-object and confirm their choice of location with a button-press. After the button-press, feedback was given to the participant via a smiley-face displayed on the screen that was either green (if the error distance between the correct location and the participant’s response was below 20 vm), or yellow (for error distances between 20 and 30 vm), or red (for error distances larger than 30 vm). If the error distance was larger than 20 vm, the target object reappeared and had to be collected, allowing for (re-)encoding of its correct location. After each trial, the participant was automatically transported to a random position within the virtual room before the next trial started.

Participants were explicitly instructed that the main goal during the task was to complete as many trials as possible while attempting to always get green smiley face feedback (i.e., as a response to error distances below 20 vm), but not to focus on being as accurate as possible (i.e., avoiding to spend time on fine tuning of their position at presumed target locations). The order of trials was pseudo-randomized, but the same order was used for all participants. Movement speed was constantly set to 15 vm per second, and it was not possible to make translational and rotational movements at the same time (i.e., it was not possible to walk curves but just straight lines). Rotation speed was constantly set to 50 deg/s. Height of the virtual camera was set to 1.7 vm. The object-location memory task was developed using the WorldViz Vizard 5.1 Virtual Reality Software (WorldViz LLC, http://www.worldviz.com).

In order to learn step-by-step how to navigate a virtual environment using a joystick and how to perform the object-location memory task, all participants underwent an extensive preparatory session on a separate day before fMRI scanning. During the preparatory session, participants first got familiarized with joystick control by completing several training tasks in a different virtual environment, before they were familiarized with the procedure of the object-location memory task by performing several trials in a parallel task version. Next, participants performed the object-location memory task in the actual test version of the task (the same one they would later navigate during the fMRI scanning-session, including the same target objects and object locations). To ensure that each participant learned the target object locations with the required accuracy, this learning procedure continued until a participant identified each target object’s location at least two times in a row with an error distance below 20 vm and until they had received at least eight green smiley faces in a row. On the day of scanning, to ensure that participants still remembered the location of the target objects, they first underwent another short block of trials outside the scanner, until they received five green smiley faces in a row. Then, each participant completed four runs of fMRI scanning while performing the object-location memory task. Each fMRI scanning run had a duration of 16 minutes.

#### MRI scanning parameters

MRI data were acquired on a 3T Siemens Magnetom Prisma scanner equipped with a 64-channel phased array head coil. All sequences described below utilized parallel imaging with a GRAPPA acceleration factor of 2.

During the object-location memory task, T2^∗^-weighted functional images were recorded with a partial volume echo-planar imaging (EPI) sequence with the following parameters: repetition time (TR) = 1500 ms, echo time (TE) = 30 ms, slice thickness = 2 mm, in-plane-resolution = 2 × 2 mm, number of slices = 24, field of view = 216 mm, flip angle = 80°, slice acquisition order = interleaved. Slices were oriented parallel to the long axis of the hippocampus.

To facilitate an accurate co-registration of entorhinal cortex masks to partial volume EPI images, a whole brain EPI image was acquired with the following parameters: TR = 6000 ms, TE = 30 ms, slice thickness = 2 mm, in-plane-resolution = 2 × 2 mm, number of slices = 84, field of view = 216 mm, flip angle = 90°, slice acquisition order = interleaved. Slices were oriented parallel to the long axis of the hippocampus.

For manual delineation of the entorhinal cortex, a high-resolution T2-weighted structural image was acquired using a hyper echo turbo-spin-echo (TSE) sequence with the following parameters: TR = 6000 ms, TE = 71 ms, slice thickness = 2 mm, in-plane-resolution = 0.5 × 0.5 mm, number of slices = 64, field of view = 224 mm, flip angle = 120°, slice acquisition order = interleaved. Slices were oriented orthogonal to the long axis of the hippocampus.

A structural T1-weighted image with isotropic resolution was acquired using an MPRAGE sequence with the following parameters: TR = 2500 ms, TE = 2.82 ms, inversion time (TI) = 1100 ms, slice thickness = 1 mm, in-plane-resolution = 1 × 1 mm, number of slices = 192, field of view = 256 mm, flip angle = 7°.

Moreover, a gradient-multi-echo sequence was acquired, which is not discussed here further.

#### fMRI data preprocessing

Functional images were realigned and smoothed with a 5 mm full-width-half-maximum Gaussian kernel using SPM12 (http://www.fil.ion.ucl.ac.uk/spm/). In order to avoid spatial distortions or interpolation errors in the data resulting from normalization to a standard template, we did not apply normalization but images were further analyzed in each participant’s native space.

#### Correction for head movement during scanning

Correction for head movement was performed at two levels: First, we applied the realignment algorithm of SPM12, which is specifically designed to account for head movement during fMRI scanning. This algorithm corrects for motion-related linear or angular displacement of scan images. Second, we included movement parameters for each scan volume (as calculated by the realignment algorithm) as regressors of no interest in every GLM that was carried out in order to calculate grid-cell-like representations. This approach corrects for movement-related signal artifacts (i.e., spin-history effects), so that other regressors in the GLM (e.g., like regressors testing for the 6-fold symmetric modulation) are not affected by movement-related changes of the BOLD signal.

#### Entorhinal cortex region of interest masks

Anatomical masks of the entorhinal cortex were traced manually on each participant’s T2-weighted image using ITK-SNAP (http://www.itksnap.org/). For manual delineation of the entorhinal cortex (Figure S1A), we followed the segmentation protocol of Berron et al. [48]. After manual delineation, region of interest (ROI) mask images were created using ITK-SNAP.

Together with the T2-weighted image, ROI mask images were co-registered to the participant’s EPI data. Given that the EPI images were only partial volume slabs (see Figure S1B, blue), the process of co-registering T2 images (Figure S1B, grayscale) to the partial volume EPI images is non-trivial, as co-registration can only be computed on a relatively small portion of overlapping brain tissue between the two different image modalities (see Figure S1B, red). Co-registration was therefore performed via one whole brain EPI image, in order to avoid co-registering images with little overlap of brain tissue, and consequently allow for a more accurate co-registration. This whole brain EPI image had similar imaging parameters as the partial volume EPI images, but a considerably higher TR to enable whole brain coverage. Co-registration of the T2 image to the partial volume EPI images included two separate steps: In a first step, the T2 image (together with the ROI masks) was co-registered to the whole brain EPI image. This co-registration step has the advantage that co-registration is facilitated by considerably more overlapping brain tissue between the two image modalities (i.e., T2 and whole brain EPI), as compared to directly co-registering the T2 image to the partial volume EPI images. In a second step, the whole brain EPI (together with the T2 image and ROI masks) was co-registered to the partial volume EPI images. This second co-registration step again has the advantage of considerably more overlap between whole brain EPI and partial volume EPI images. Furthermore, whole brain EPI and partial volume EPI images share similar imaging parameters and therefore have widely similar properties, which also facilitates a more accurate co-registration.

#### Analysis of grid-cell-like representations

For the analysis of grid-cell-like representations, we used the Grid Code Analysis Toolbox (GridCAT) with MATLAB 2016b (The MathWorks, Natick, MA, USA). The MATLAB source code that was used to carry out this analysis is freely and openly available [18]. The analysis followed the procedure of Doeller et al. [15]. First, we partitioned the four fMRI scanning runs into two data halves, using the first half of each scanning run as the estimation dataset and the second half of each scanning run as test dataset. Then, we estimated voxel-wise grid orientations by fitting the estimation data to a first general linear model (GLM1). This model included two parametric modulation regressors for all translation events, using sin(α_t_^∗^6) and cos(α_t_^∗^6), respectively, where α_t_ represents an event’s translation direction within the virtual environment. We then calculated the mean grid orientation (φ) within entorhinal cortex voxels, by averaging the beta estimates (β_1_ and β_2_) associated with the two parametric modulation regressors over all voxels in the left and right entorhinal cortex, and submitting the resulting two values to: arctan[mean(β_1_)/mean(β_2_)]/6. Then, the remaining half of the data (test dataset) was modeled in a second general linear model (GLM2). This model included a parametric modulation regressor for all translation events, calculated by taking each event’s translation direction (α_t_), and determining its difference from the mean grid orientation (φ) by calculating cos[6^∗^(α_t_ – φ)]. Finally, the magnitude of grid-cell-like representations in entorhinal cortex was quantified by the average parametric modulation regressor’s parameter estimates within entorhinal cortex voxels. Based on these model specifications, magnitudes of grid-cell-like representations are expected to be positive for changes in mean grid orientation of less than ± 15° between data halves, or negative for changes of more than ± 15°, given that grid orientations range from 0° to 60° and therefore the maximally detectable change in grid orientation is 30°.

Importantly, in both GLM1 and GLM2, only translation events but not other events (such as stationary periods) were parametrically modulated by sine/cosine regressors with respect to their translation direction, in order to ensure that the calculation of voxel-wise grid orientations and magnitudes of grid-cell-like representations was only based on modeling events during which participants performed translational movements in the virtual environment.

In all GLMs, we included as regressors of no interest head motion parameters (x, y, z, yaw, pitch, and roll) derived from realignment in SPM, the unused grid events (i.e., the parametrically modulated translation events for GLM2 when fitting GLM1, and vice-versa), and the feedback phase in the object-location memory task. Very short translation events (duration < 1 s) were not modeled in the GLMs.

#### Analysis of representational stability

The ability to detect grid-cell-like representations in fMRI can be affected by the homogeneity of the estimated grid orientations across voxels within an ROI (i.e., spatial stability). Specifically, if all voxels within an ROI provide a different orientation value (i.e., high spatial instability), then the resulting mean grid orientation would be random, and the coding of translation events in GLM2 (in which translation events are modeled with respect to the deviation between their translation direction and the mean grid orientation), would then be arbitrary, resulting in a reduced grid-cell-like representation magnitude. Alternatively, an inability to detect grid codes in the fMRI signal could result from instability of the estimated grid orientations over time (i.e., temporal stability).

Calculation of metrics for spatial and temporal stability of each participant’s grid-cell-like representations followed the methods described in Kunz et al. [16] and Stangl et al. [18]: In order to obtain a metric of spatial stability, we calculated the coherence of estimated voxel-wise grid orientations between all entorhinal cortex voxels by submitting the orientation values to Rayleigh’s test for non-uniformity of circular data. Spatial stability is then statistically expressed by the resulting Rayleigh’s z-value (with higher z-values indicating higher spatial stability). To calculate temporal stability, we first estimated voxel-wise grid orientations for each half of a scanning run separately. For each individual voxel, we then compared the orientation between first and second data half, and classified each voxel’s orientation as “stable” if the orientations were within ± 15° of one another. Temporal stability is then indicated by the average proportion of stable voxels within entorhinal cortex across all scanning runs. In a separate analysis using an alternative measure of temporal stability, we calculated the absolute change in grid orientation between data halves for each voxel separately, and then calculated the average change in orientation across all entorhinal cortex voxels.

#### Control analyses

Given the different data amounts of translation data between young and older adults, magnitudes of grid-cell-like representations in young adults were re-analyzed using only a proportion of their translation data. Specifically, the amount of translation data we acquired from older adults was on average 80.16 percent of the data from young adults. Two different ways of reducing the data were implemented in two separate control analyses: First, we shortened each scanning run of young adults by discarding the last part of each run so that only 80.16 percent of translation data were taken into account. Second, we shortened each individual translation phase and modeled only the first 80.16 percent of each phase in a participant’s GLMs to analyze grid cell-like representations.

Since there is evidence that task performance in fMRI studies might modulate age differences in brain activation [49], we also checked whether accuracy in the object-location memory task had an impact on grid-cell-like representations. For these control analyses, task performance was quantified by each participant’s average error distance across all trials in the object-location memory task.

It has been shown that a reduction in entorhinal cortex volume might occur during pathological and healthy aging [13]. Such a volume reduction in older adults could have driven the age effect in grid-cell-like representations, as a reduced number of entorhinal cortex voxels might have led to a less reliable estimation of grid orientations and, consequently, to reduced temporal representational stability. We therefore checked whether entorinal cortex volume might account for differences in grid-cell-like representation magnitudes between age groups. Entorhinal cortex volume was quantified by the number of entorhinal cortex voxels in each participant’s individual T2-weighted structural image.

We also tested whether the lack of grid-cell-like representations in older adults might be a general effect of lower fMRI signal quality in entorhinal cortex of older adults. As an indicator for signal quality, we calculated the temporal signal-to-noise ratio (tSNR) within entorhinal cortex of each participant. tSNR was quantified by the mean signal within entorhinal cortex divided by the standard deviation of this signal over time.

In order to test whether head movement during fMRI scanning might account for differences in grid-cell-like representation magnitudes between age groups, we calculated each participant’s average linear and angular displacement per scan volume. Motion parameters were extracted from the realignment procedure in SPM12, which specifically quantifies both linear and angular head movement during scanning. Individually for each participant, we calculated the sum of displacement per scan in all three linear dimensions (x + y + z) and then calculated the average linear displacement per scan across the fMRI time series. The same procedure was performed for angular displacement (yaw + pitch + roll).

#### Path integration task

In commonly used path integration tasks for humans, such as the triangle completion task [29, 50], participants traverse a path and only estimate the distance and direction to the starting location at the end of the path. In the current study, however, we used a task in which participants were asked at three different points along the path to estimate the distance and direction to the path’s starting point (Figure 3A). Multiple distance and direction judgments per path were used for two reasons: First, it results in a larger number of data points (i.e., participant responses) in a similar amount of time, enabling a more reliable estimation of path integration errors. Second, responses from multiple points along the path can allow for a more precise estimation of path integration errors. Specifically, when complex paths are used, a participant may become disorientated as they move along the path, and the chances of this occurring increase with the distance traversed. When only one response is collected at the end of the path, as per the traditional triangle completion task, the participant’s estimate would be random and not provide a valid quantification of path integration performance. In contrast, our task samples from multiple points along the path meaning that, even if the participant has become disorientated at the path’s end point, there are still other data points earlier in the path that provide more accurate estimates of path integration performance.

In this task, participants had to keep track of their own position during movement along pre-defined curved paths (Figure 3A), while a head-mounted display (HMD; Oculus Rift Development Kit 2, Oculus VR LLC, http://www.oculus.com) was positioned on the participant’s head, so that they could not see anything outside the HMD. The task was implemented in two different modalities: In the “body-based” modality (Figure 3B), no visual input was shown via the HMD (like in complete darkness), and participants could only use body-based self-motion cues, such as proprioceptive and vestibular representations, as well as motor efference copies, that are produced during movement [51]. Participants held a wooden stick and were guided by the experimenter along a path. At each of three stopping points along the path, the distance to the starting point had to be estimated verbally in meters and centimeters, and participants turned their body on the spot to indicate the orientation to the starting point, which was measured by the built-in gyrometer of the HMD. In the “visual” modality (Figure 3C), participants sat stationary on a chair, while they saw a virtual environment via the HMD. This virtual environment did not contain any landmark cues but only showed a floor texture consisting of 500 white dots on a black ground plane. Each dot appeared at a random position within the field of view and disappeared after a random duration of 1 to 3 s, before it reappeared at a different location. Consequently, these so-called “limited lifetime dots” provided optic flow information, while their limited lifetime prevented the use of fixed reference points. Automated movement along a path was shown from first-person perspective, and on each of three stopping points along the path, participants estimated the distance to the path’s starting point verbally in meters and centimeters, and indicated the presumed orientation by turning their view in the virtual environment to the left or right using a joystick.

Prior to the task, participants received written information about the task, and completed several practice paths in both modalities. During the task, participants wore earmuffs in order to prevent them from hearing any background sounds. Also, they were instructed to immediately inform the experimenter if they noticed any external cues that could help them to orient during the task (such as hearing, seeing, feeling or smelling something).

There were eight different pre-defined paths (Figure S3C), and each path was performed two times per modality. The order of paths was pseudo-randomized, but the same order was used for all participants. Consequently, each participant completed 32 paths in total (16 paths per modality). Paths in different modalities were intermixed, but there were always at least three different paths between two occurrences of the same path (irrespective of the modality).

Coordinates for each of the eight pre-defined paths were defined as follows: First, a 3-legged path was created that comprised three distances and two turning angles between them (Figure S3A). Each distance was either 2 m, 3 m, 4 m, or 5 m, and each angle was either 55°, 80°, or 105° to the left or to the right. Various combinations of distances and angles were used, that fit into a rectangular area of approximately 10 × 6 m (given by the size of the room in which the experiment took place). On the basis of these 3-legged paths, we then created curved paths without corners by using the cscvn-function of MATLAB’s curve fitting toolbox to calculate a natural interpolated cubic spline curve passing through the turning points of the 3-legged path. Directions (left versus right) of the two turning angles per path were counter-balanced between the different paths (Figure S3C). The experimenters ensured that participants did not see the real physical dimensions of the room and the paths before and during the experiment.

Participants completed the 32 paths in two blocks of 16 paths each. In the middle of each block, participants completed 4 so-called “standardization paths,” which were needed for data analysis in order to correct each participant’s distance estimate for their ability to use a correct estimate of a meter/centimeter when verbalizing their response (see “Path integration data analysis” section). The procedure during a standardization path was similar as during a normal path, but a standardization path had only one starting point and one stopping point, which were connected by a straight line, and participants had to estimate the distance between starting and stopping point. Two different distances (3 m and 9 m) had to be estimated both in body-based and visual modality, in the following order: 9m_visual, 3m_body-based, 9m_body-based, 3m_visual.

After completing the task, participants filled out a form in which they were asked whether they noticed any external cues that could have helped them to orient during the task (such as hearing, seeing, feeling or smelling something), but no participant reported such confounding sources of information. Further, all participants were asked whether they had recognized that some paths were repetitions of each other, but no participant did.

The path integration task was developed using the Unity game engine 5.3.5 (Unity Technologies, http://www.unity3d.com).

#### Path integration data analysis

At every stopping point of a path, participants had to estimate the distance to the path’s starting point verbally in meters and centimeters. However, due to these verbal estimates, the responded distance can be influenced not only by the participant’s path integration performance, but might also be confounded by their ability to use a correct estimate of a meter/centimeter when verbalizing the response. For example, a participant with perfect path integration performance might still give suboptimal answers, just because they might have a relatively divergent perception of what they consider to be “a meter” (which might be unrelated to path integration performance per se). To get a “standardized” measure of path integration performance, we corrected each participant’s responses for their individual ability to verbalize their distance estimate in meters/centimeters, and use their individual measure of what they perceive as a meter. Separately for each modality, we created an indicator of what each individual participant considered to be a meter by taking the responded distances from standardization paths (in which just a straight distance without any curves had to be estimated), and calculating:

$$\text{f}={\text{d}}_{\text{correct}}/{\text{d}}_{\text{response}}$$

where d_correct_ is the correct distance of the standardization path (either 3 m or 9 m), d_response_ is the responded distance, and f is the resulting correction factor. Responded distances from standardization paths of the same distance (3 m versus 9 m) and modality (body-based versus visual) were averaged. For each participant, this led to two different correction factors per modality, one each for shorter distances (derived from the 3 m standardization path) and longer distances (derived from the 9 m standardization path). These factors were used to standardize the distance estimates this participant reported at normal paths: Whenever the participant’s response distance of a normal path was between 0 m and 6 m, the response was multiplied with the correction factor for shorter distances, whereas response distances larger than 6 m were multiplied with the correction factor for longer distances.

At each stopping point, the responded distance (multiplied with the individual participant’s correction factor) and orientation was used to calculate the “presumed starting point.” The x and y coordinates of the presumed starting point according to the participant’s response were calculated by:

$$\begin{array}{l} {\text{x}}_{\text{presumedStart}}={\text{x}}_{\text{stop}}+{\text{d}}_{\text{standardized}}*\cos\left({\text{ori}}_{\text{response}}\right)\\ {\text{y}}_{\text{presumedStart}}={\text{y}}_{\text{stop}}+{\text{d}}_{\text{standardized}}*\sin\left({\text{ori}}_{\text{response}}\right) \end{array}$$

where d_standardized_ is the standardized response distance, and ori_response_ is the responded orientation. x_stop_ and y_stop_ are coordinates of the stopping point, x_presumedStart_ and y_presumedStart_ are the resulting coordinates of the presumed starting point.

In order to determine the path integration error for a given stopping point, the Euclidean distance between the presumed starting point (according to the participant’s response at this respective stopping point) and the previously presumed starting point (according to the response at the previous stopping point) was calculated. Note that the previously presumed starting point at stopping point 1 is the correct starting point of the path. Consequently, this measure of the path integration error reflects only the incremental error that occurred on the latest path segment before the stopping point, but does not include the error that occurred on earlier segments of the same path (Figure S3B). Therefore, this method of calculating the path integration error allows, for each individual participant, to aggregate all error measures from all available stopping points, because each path integration error measure includes only the incremental (i.e., unique) error contribution of one path segment. Finally, each participant’s individual path integration error was calculated by averaging path integration errors across all stopping points, separately for body-based and visual modality. Path integration performance was calculated by 1 / path integration error.

#### Neuropsychological tests

Neuropsychological test scores of older adults were taken from a database of the German Center for Neurodegenerative Diseases Magdeburg. These neuropsychological tests were carried out within 12 months before or after participants took part in the path integration and fMRI experiment. One older adult did not complete neuropsychological testing. For this participant, only the magnitude of grid-cell-like representations and demographic variables (age, sex), but not neuropsychological test scores were used in the multiple linear regression analysis. For the remaining 20 older adults, the following neuropsychological test scores were used: Self-reported spatial abilities, measured by the Santa Barbara Sense of Direction Scale (SBSOD [52]). Spatial working memory, measured by the Corsi block-tapping task [53]. Spatial attention, measured by the subtest “visual scanning,” and general working memory, measured by the subtest “working memory” of the Testbattery for Attentional Performance (TAP, Zimmermann P. & Fimm B., Psychologische Testsysteme, Herzogenrath, Germany). Processing speed, measured by the Digit Symbol Substitution Test (DSST) of the Wechsler Adult Intelligence Scale (WAIS-IV, Wechsler D., Pearson Assessment, San Antonio, TX, USA). Cognitive status, measured by the Montreal Cognitive Assessment (MoCA [45]).

### Quantification and Statistical Analyses

All statistical analyses were carried out using MATLAB 2016b and the Statistics and Machine Learning Toolbox for MATLAB 2016b. Correlation values, where given, are Pearson correlations. Error bars in Figures indicate standard-errors of the mean (SEM). Statistical analyses were performed using a significance threshold of p < 0.05.

We used one-sample t-tests in order to test whether magnitudes of grid-cell-like representations (6-fold model) or symmetrical models with different periodicities (5-/7-fold) were significantly different from zero. In young adults, paired-sample t-tests were used to compare magnitudes of grid-cell-like representations in the reduced translation data models versus the full data model. Group comparisons between young and older adults were carried out using two-sample t-tests.

In order to divide the group of young adults into three subgroups with low, middle, and high magnitudes of grid-cell-like representations, a cluster analysis was applied on these magnitudes, using the k-means clustering function in MATLAB. Centroid starting locations (seeds) were defined by the mean magnitude of grid-cell-like representations for the 6 lowest, the 6 middle, and the 6 highest magnitudes of young adults, respectively.

Further details on statistical analyses are given in the Results and Discussion section and in the STAR Methods section.

## Supplemental Information

Document S1. Figures S1–S3

Document S2. Article plus Supplemental Information
