# Supplementary material for: Compromised Grid-Cell-like Representations in Old Age as a Key Mechanism to Explain Age-Related Navigational Deficits
Source: Curr Biol. 2018 Apr 2;28(7):1108–1115.e6. doi: 10.1016/j.cub.2018.02.038 (PMC5887108; doi:10.1016/j.cub.2018.02.038)
Supplement: Document S1. Figures S1–S3 [file mmc1.pdf]

Current Biology, Volume *28*

Supplemental Information

Compromised Grid-Cell-like Representations in Old Age as a Key Mechanism to Explain Age-Related Navigational Deficits

Matthias Stangl, Johannes Achtzehn, Karin Huber, Caroline Dietrich, Claus Tempelmann, and Thomas Wolbers

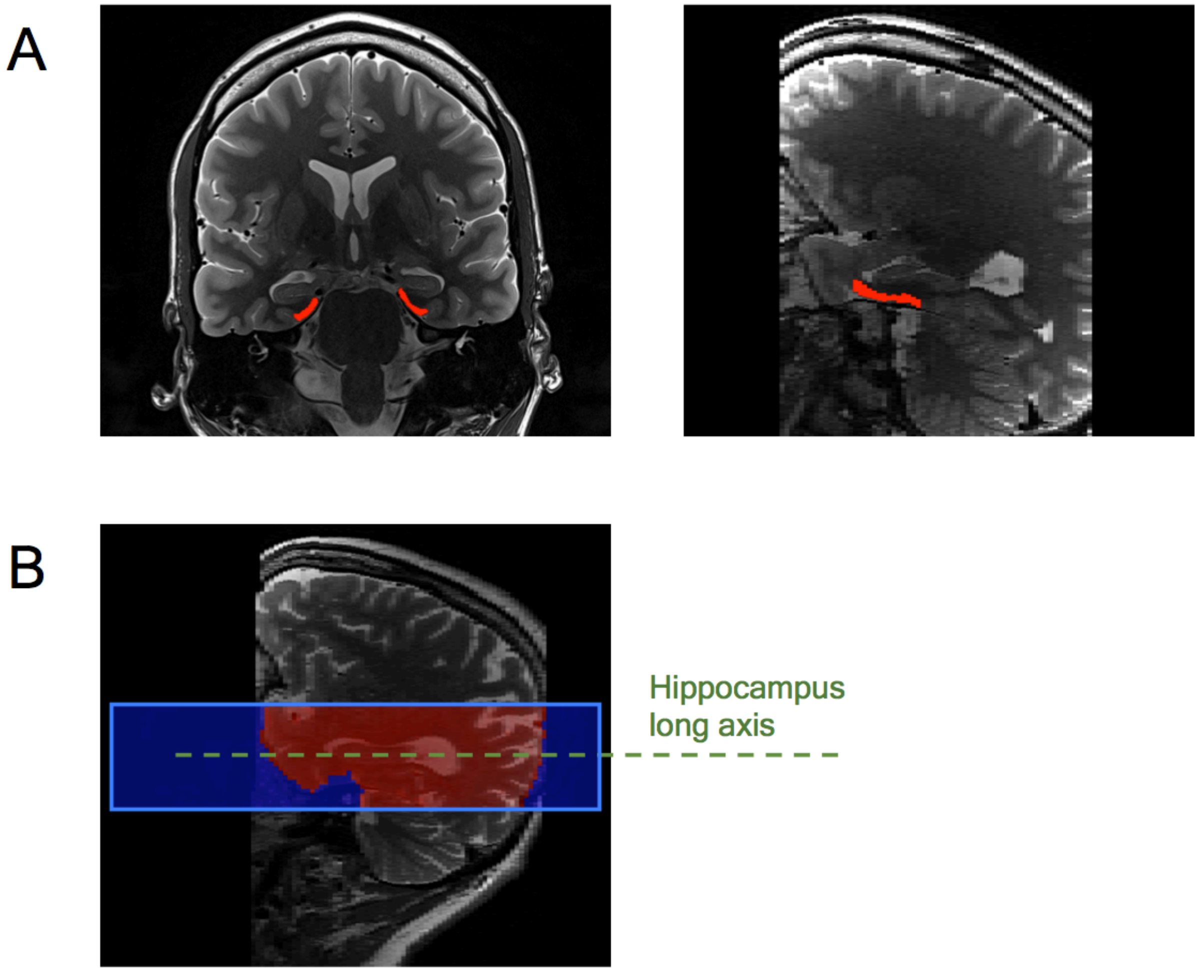

Figure S1. Related to Figure 2 and STAR Methods.

(A) Example mask for the bilateral entorhinal cortex (red) of one participant, shown on the participant's individual T2-weighted structural image in coronal (left) and sagittal (right) view.

(B) Partial volume size and orientation of an EPI image (blue) shown on the T2-weighted structural image (grayscale) of one participant in sagittal view. Overlap between partial volume EPI and T2 image is shown in red. Slices of the T2 image were acquired orthogonal to the long axis of the hippocampus (indicated by the green dashed line), whereas slices of partial volume EPI images were oriented parallel to the long axis of the hippocampus.

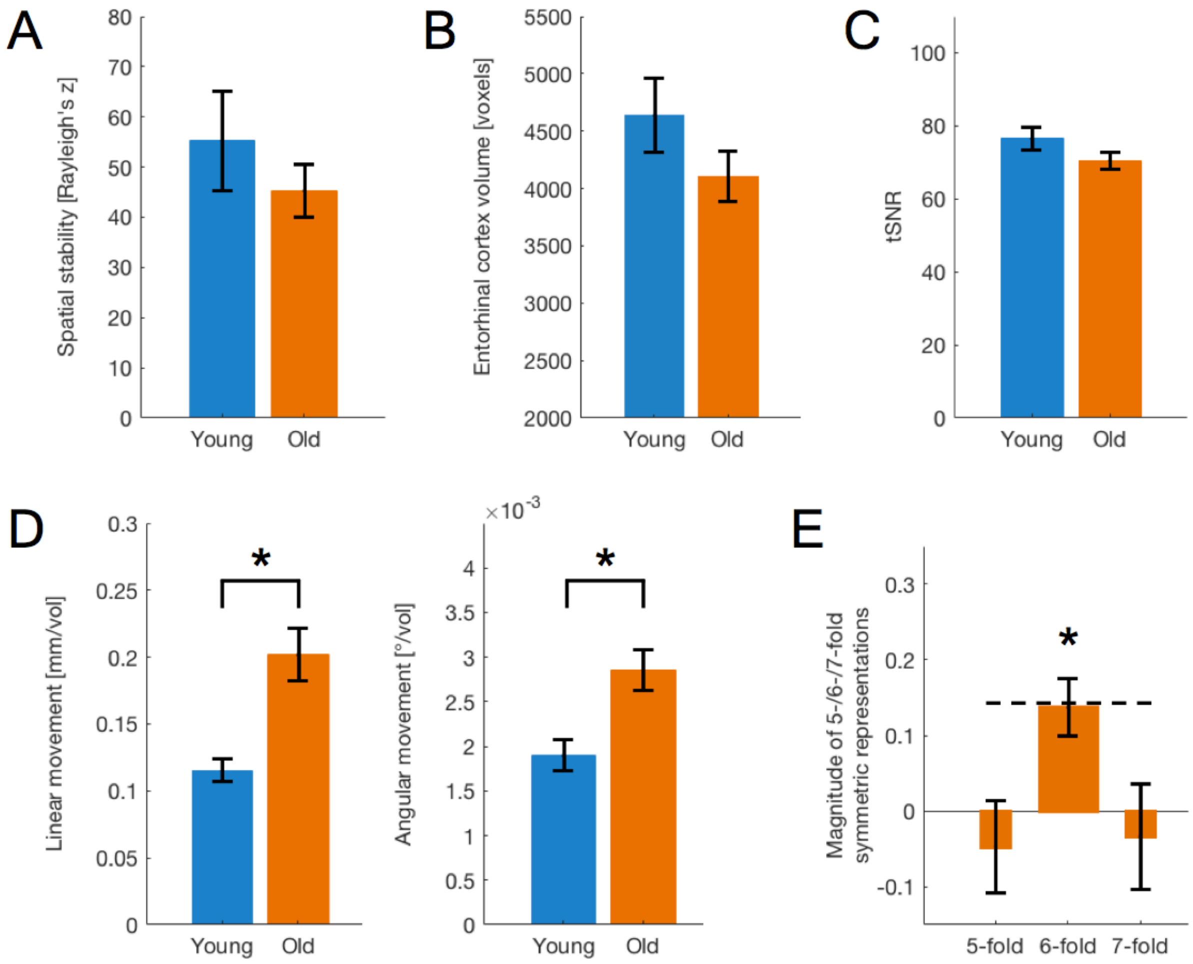

Figure S2. Related to Figure 2 and STAR Methods.

(A) Spatial stability of grid-cell-like representations in entorhinal cortex voxels was not significantly different between young and older adults.

(B) Volume size of entorhinal cortex was not significantly different between young and older adults. Volume size was measured by the number of entorhinal cortex voxels in each participant's individual T2-weighted structural image.

(C) Temporal signal-to-noise ratio (tSNR) in entorhinal cortex was not significantly different between young and older adults. tSNR was quantified by the mean signal within entorhinal cortex divided by the standard deviation of this signal over time.

(D) Older adults showed more linear and angular head movement during scanning. Head movement was quantified by mean displacement per scan volume.

(E) Magnitudes of grid-cell-like representations (6-fold) and control models (5-/7-fold) shown for a subgroup of older adults (n = 10) with low path integration errors in the body-based modality. Dashed line indicates the mean grid-cell-like representation magnitude of young adults. Units are parameter estimates.

Error bars indicate SEM. *$p < 0.05$

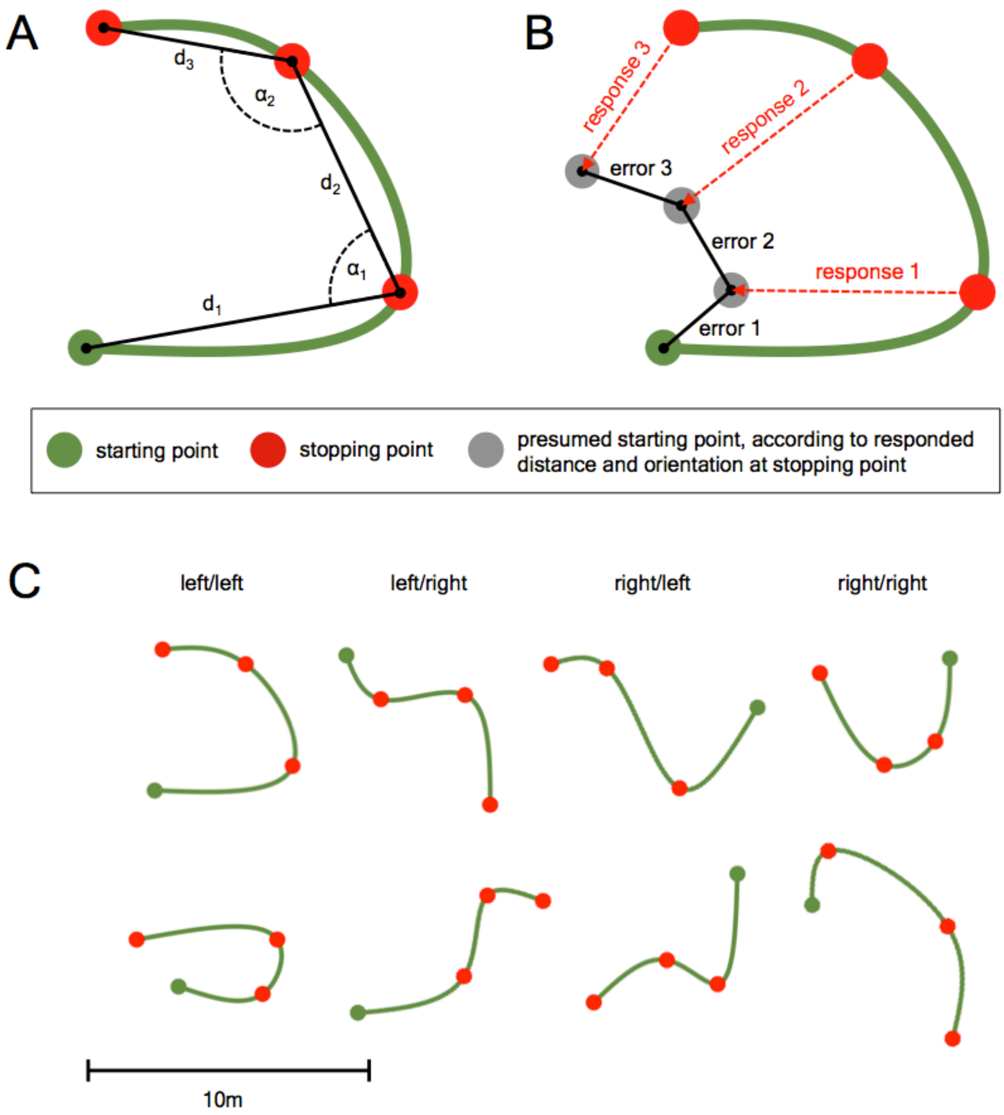

Figure S3. Related to Figure 3 and STAR Methods.

(A) Each path was created by first defining a 3-legged path (black solid lines) consisting of three distances (d_1, d_2, d_3) and two turning angles between them (α_1, α_2). Participants walked along a curved version of this 3-legged path that had no corners and was created by a natural interpolated cubic spline curve passing through the path's turning points (green line).

(B) Path integration error was calculated for each stopping point separately (error 1, error 2, error 3). For a given stopping point, it was calculated by the Euclidean distance between the presumed starting point according to the participant's response at this respective stopping point, and the previously presumed starting point according to the response at the previous stopping point. Path integration errors therefore reflect only the incremental error that occurred on the last path segment before the stopping point, but do not include errors that occurred on earlier segments of the same path.

(C) Overview over the eight paths that were used in the experiment. Turning directions (left vs. right) of the two turning angles per path were counter-balanced between the different paths, leading to two paths with each of the following combinations: left/left (column 1), left/right (column 2), right/left (column 3), right/right (column 4).
